# Striatal Circuits as a Common Node for Autism Pathophysiology

**DOI:** 10.3389/fnins.2016.00027

**Published:** 2016-02-09

**Authors:** Marc V. Fuccillo

**Affiliations:** Department of Neuroscience, Perelman School of Medicine, University of PennsylvaniaPhiladelphia, PA, USA

**Keywords:** autism spectrum disorders, dorsal striatum, nucleus accumbens (NAcc), mouse models, synaptic transmission, circuit

## Abstract

Autism spectrum disorders (ASD) are characterized by two seemingly unrelated symptom domains—deficits in social interactions and restrictive, repetitive patterns of behavioral output. Whether the diverse nature of ASD symptomatology represents distributed dysfunction of brain networks or abnormalities within specific neural circuits is unclear. Striatal dysfunction is postulated to underlie the repetitive motor behaviors seen in ASD, and neurological and brain-imaging studies have supported this assumption. However, as our appreciation of striatal function expands to include regulation of behavioral flexibility, motivational state, goal-directed learning, and attention, we consider whether alterations in striatal physiology are a central node mediating a range of autism-associated behaviors, including social and cognitive deficits that are hallmarks of the disease. This review investigates multiple genetic mouse models of ASD to explore whether abnormalities in striatal circuits constitute a common pathophysiological mechanism in the development of autism-related behaviors. Despite the heterogeneity of genetic insult investigated, numerous genetic ASD models display alterations in the structure and function of striatal circuits, as well as abnormal behaviors including repetitive grooming, stereotypic motor routines, deficits in social interaction and decision-making. Comparative analysis in rodents provides a unique opportunity to leverage growing genetic association data to reveal canonical neural circuits whose dysfunction directly contributes to discrete aspects of ASD symptomatology. The description of such circuits could provide both organizing principles for understanding the complex genetic etiology of ASD as well as novel treatment routes. Furthermore, this focus on striatal mechanisms of behavioral regulation may also prove useful for exploring the pathogenesis of other neuropsychiatric diseases, which display overlapping behavioral deficits with ASD.

## A circuit hypothesis for autism spectrum disorder pathophysiology

### Moving from a clinical to molecular characterization of autism spectrum disorders

The earliest clinical descriptions of autism highlighted two symptom domains, focusing on social behaviors and the regulation of motor output. Kanner's seminal article “Autistic Disturbances of Affective Contact” carefully described the profound social deficits of his patients, concluding “these children have come into the world with innate inability to form the usual biologically provided affective contact with people…” (Kanner, 1943). Shortly thereafter, in his “‘Autistic Psychopathy’ in childhood,” Asperger documented a range of abnormalities in behavioral control (“most conspicuous were his stereotyped movements: he would suddenly start to beat rhythmically on his thighs, bang loudly on the table, hit the wall…”), motor performance (when trying a “particular physical exercise, his movements would be ugly and angular”) and goal-directed actions (“…drives and instincts are often severely perturbed. This is shown in the failure of instinctive situational adaptation…”; Asperger, 1944). The detailed clinical observations from these papers highlighted the diversity of behavioral presentations in autism and demonstrated the extensive comorbidity between symptoms in the social and motor control domains. Nearly 70 years later, the fruits of the genetic revolution are beginning to reveal molecular abnormalities contributing to the behaviors originally observed by Kanner and Asperger (Krumm et al., 2014; Willsey and State, 2015). In an attempt to generate a coherent pathophysiological hypothesis of autism spectrum disorders (ASD) that considers both the diversity of implicated proteins as well as the range of observed behavioral phenotypes, I will focus on deficits within striatal circuitry. First I will examine how diverse theoretical concepts of striatal function may relate to key ASD symptom domains. Next, I provide evidence from both the clinical and experimental literature that suggests a pattern of core striatal dysfunction in ASDs. Finally, I will explore why the striatum might occupy such a central place in autism pathophysiology and how we might use this information to refocus our treatment endeavors.

### The implications of studying circuit dysfunction in neuropsychiatric disease

From the outset, it is worthwhile considering the utility of exploring nervous system disease pathophysiology from the vantage of neural circuit dysfunction. This approach seeks to uncover alterations in defined, reproducibly interconnected sets of neurons that are responsible for discrete behavioral phenomenon seen in neuropsychiatric diseases such as schizophrenia (Spellman and Gordon, 2015), obsessive-compulsive disorder (Ahmari and Dougherty, 2015; Monteiro and Feng, 2016), and mood disorders (Fox and Kalin, 2014; Lammel et al., 2014). It does not attempt to link any of these complex diseases to a single region, as the mammalian brain is a massively interconnected structure whose full functional output certainly relies on coordinated and parallel processing between multiple areas. Nonetheless, the approach does seek to uncover specific circuit nodes for disease pathophysiology—neuronal connections that are uniquely vulnerable to genetic or environmental insult which also have a key role in regulating behavioral output. Alterations in these nodes may represent an initiating event that triggers subsequent downstream adaptations that together become the driver of abnormal behavior. My focus on the involvement of striatal dysfunction in this review by no means precludes the involvement of other brain regions. Rather, given the intersection between motor and cognitive abnormalities seen in ASD, the prefrontal cortex and cerebellum may represent equally susceptible terrain for the physiological alterations that drive behavioral changes (Fatemi et al., 2012; Martinez-Sanchis, 2014; Wang et al., 2014; Bicks et al., 2015; Chmielewski and Beste, 2015). The potential importance of these systems and their interactions with basal ganglia circuits will be considered in detail later.

### The basal ganglia: an evolutionarily conserved neural circuit for weighing costs

**Figure 1:**
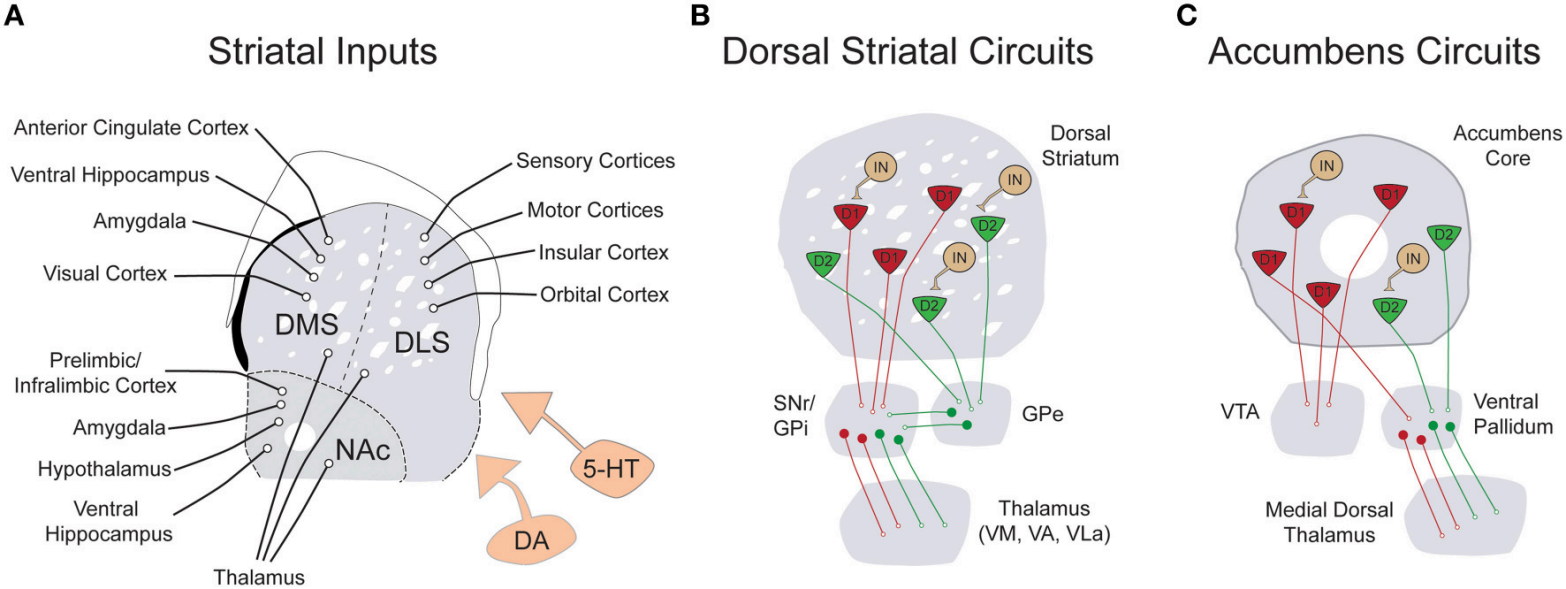
**Simplified schematic of the input and local connectivity of the mammalian basal ganglia. (A)** Diagram of known excitatory inputs to distinct striatal sub-regions including dorsomedial striatum (DMS), dorsolateral striatum (DLS), and nucleus accumbens (NAc). All striatal sub-regions are under dopaminergic and serotonergic neuromodulatory control (orange). **(B)** Diagram of major dorsal striatal cell types and their downstream connectivity within basal ganglia circuits. In contrast to striatal inputs, nearly all cell types within the basal ganglia are inhibitory. The striatum is comprised of D1R+ (red) and D2R+ (green) medium spiny neurons (MSNs), as well as a smaller population of local circuit interneurons (tan). The direct pathway projection of D1R+ MSNs is diagrammed in red and the indirect pathway projection of D2R+ MSNs is drawn in green. **(C)** Diagram of the major cell types and connectivity of the nucleus accumbens core. Note that in contrast to the strict divergence of D1R+ and D2R+ MSNs in the dorsal striatum, D1R+ MSNs of the nucleus accumbens project both to the ventral tegmental area and the ventral pallidum.

**Figure 2:**
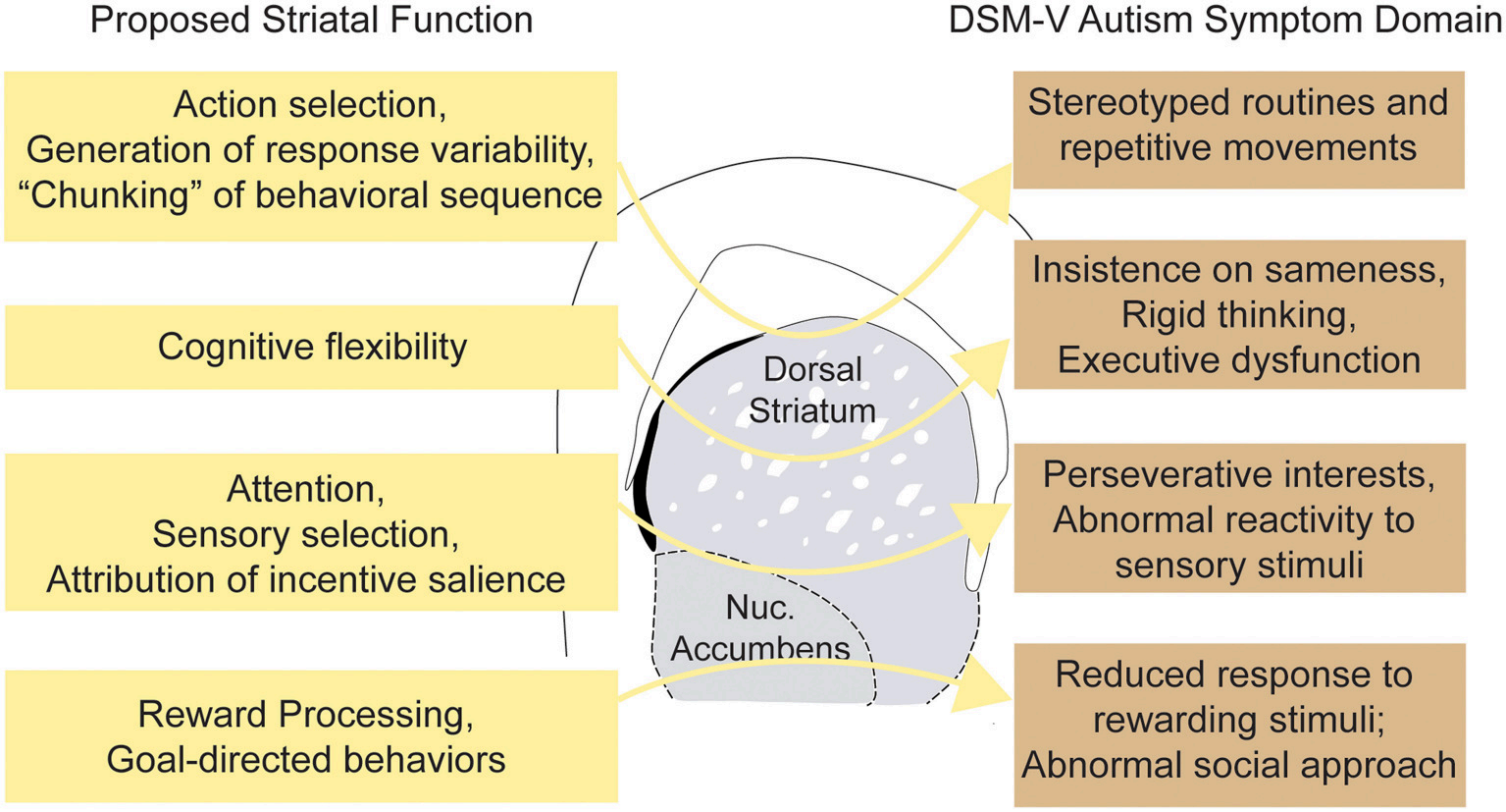
**Striatal dysfunction and major autism spectrum disorder symptom domains**. Schematic illustrating hypothetical connections between abnormalities of striatal function and the varied clinical phenotypes observed in ASDs.

The striatum is the input structure of the basal ganglia, a series of interconnected subcortical nuclei first appearing in the vertebrate lineage approximately 530 million years ago (Murray et al., 2011). While the overall anatomical organization and immunohistochemical composition of this region has remained largely unchanged dating back to anamniotes, connectivity with cortical circuitry has been significantly enhanced in mammalian lineages (Medina and Reiner, 1995; Reiner et al., 1998). From its inception, the basal ganglia has likely served as an essential intermediary between an organism and its outside environment. However, what began as a relatively simple structure linking incoming sensory information to regulation of motor output, has evolved into a complex circuitry capable of computing “cost-benefit” algorithms and selecting optimally efficient actions based upon incoming sensory information, previous memories, expectations and current motivational state (Hikosaka, 1998; Daw et al., 2006; Floresco et al., 2008). The proposed functions of basal ganglia circuits are in part derived from its extraordinary anatomical organization. The striatum receives a wealth of convergent excitatory projection inputs from motor and sensory cortex, hetero-modal association areas, thalamic nuclei, hippocampus, prefrontal cortical regions, insula, and amygdala (Figure 1A; Kelley et al., 1982; Gerfen, 1984; Malach and Graybiel, 1986; Voorn et al., 2004; Pan et al., 2010). These excitatory projections diffusely synapse onto D1 dopamine receptor expressing (D1R+) and D2 dopamine receptor expressing (D2R+) medium spiny neurons (MSNs) of the dorsal striatum, which differentially project via the direct pathway to the substantia nigra pars reticulata (SNr)/internal segment of the globus pallidus (GPi or entopeduncular nucleus in rodents) and via the indirect pathway to the external segment of the globus palliudus (GPe), respectively (Figure 1B; Gerfen et al., 1990; Surmeier et al., 1993; Kreitzer and Malenka, 2008; Gerfen and Surmeier, 2011). While both circuits eventually target motor regions of thalamus, the presence of an additional inhibitory connection in the indirect pathway is thought to account for the opposing effects of striatal medium spiny neuron subtype on thalamic output. Activation of direct pathway MSNs relieves thalamic inhibition and promotes motor output while activation of indirect pathway MSNs maintains pallidal inhibition of thalamus, reducing motor output. This long-standing model for the dichotomous function of striatal MSNs on movement, based originally on clinical observations (Albin et al., 1989), has recently been confirmed by cell type-specific optogenetic interrogation (Kravitz et al., 2010). Following modulation by midbrain nuclei, thalamic neurons project back to the same regions of cortex that initially targeted striatum, providing sensory feedback control of ongoing behaviors (Bosch-Bouju et al., 2013). However, the seemingly “closed-loop” nature of these circuits is interrupted and expanded, perhaps through non-reciprocal cortico-thalamic pathways (McFarland and Haber, 2002) and spiraling dopaminergic inputs (Haber et al., 2000), to allow for iterative stages of cortico-striato-thalamic processing. In this manner, information from higher cortical areas involved in the cognitive aspects of action is transmitted to primary motor areas for the execution of specific motor output (McFarland and Haber, 2002). The functions of the basal ganglia are numerous and exist along a continuum framed on one end by sensorimotor control and on the other, by the generation of motivated and intentioned behaviors. While it is still largely speculative, extensive overlap between ASD symptomatology and striatal function seems apparent across this entire range (Figure 2).

## From motor control to motivated behaviors: diversity of basal ganglia function and its relation to ASD symptoms

### Sensory control

While early vertebrate lineages utilized a simple neural network to transform sensory information into direct motor responses, vertebrate evolution has selected circuits of increasing complexity that extract more information from the environment and effectively use it to guide motor behaviors (Murray et al., 2011). Basal ganglia circuits, developing in parallel with pallial structures and midbrain dopamine nuclei, comprise a core computational unit enabling this more sophisticated control of motor output (Stephenson-Jones et al., 2011). To achieve this, the basal ganglia had to address two areas of increasing complexity in higher vertebrates—(1) the growth of incoming information from more specialized sensory systems (sensory-selection), and (2) the expanding number of motor responses that were possible in response (action-selection). The increasing diversity and convergence of sensory inputs in developing vertebrates necessitated a robust mechanism of sensory selection to extract context-relevant information (Hikosaka, 1998). Studies on the regulation of memory-guided saccades in primate suggest the basal ganglia serves this purpose by using its inhibitory connections to gate which sensory inputs regulate collicular output, and thereby select the environmental information guiding memory-based saccades (Hikosaka and Wurtz, 1985). Alternative examples of basal ganglia-mediated sensory control may be on display when animals dynamically adjust their level of attention to salient features of the environment. In a provocative model by Krauzlis and colleagues, attention does not occur via filtering of sensory representations within the neocortex, but rather as a byproduct of neural computations made by the basal ganglia to correctly determine internal state (Krauzlis et al., 2014). Here, the basal ganglia match motivational drives and previous task history with sensory information from the external environment, in hopes of achieving the most accurate assessment of current reality from which to make future decisions. The abrogation of ongoing motor activities by salient sensory stimuli may also be mediated through basal ganglia circuits. Specifically, thalamic projections to striatal cholinergic interneurons produce a brief period of reduced cortical drive to both MSN subtypes, followed by a period of enhanced sensitivity of the indirect pathway—a key mediator of motor suppression (Ding et al., 2010). Abnormalities in sensory processing are a widespread clinical feature of ASD and frequently manifest as abnormal reactivity to sensory aspects of the environment (Gomot et al., 2006; Kwakye et al., 2011; Elwin et al., 2013). In addition, patients with autism exhibit robust deficits in the voluntary control of saccades (Minshew et al., 1999) and may use fronto-striatal neural circuits, typically reserved for higher cognitive processes, to compensate for broad sensorimotor deficiencies (Takarae et al., 2007). Finally, it will be worthwhile to explore whether dysfunction in regulating attentiveness to sensory stimuli may provide a substrate for the development of highly focused, fixated interests seen in ASD.

### Action selection and motor patterns

Basal ganglia circuits have also been hypothesized to function in action-selection, whereby a single behavioral output is selected and executed from a range of motor programs. The neural mechanisms mediating this selection process remain controversial but *in vivo* (Samejima et al., 2005; Kimchi and Laubach, 2009; Seo et al., 2012; Tai et al., 2012) and computational studies (Humphries et al., 2006; Lisman, 2014; Gurney et al., 2015) suggest the striatum and downstream basal ganglia nuclei have a central function. One hypothesis posits that distributed, synchronized extra-striatal excitation recruits specific MSN populations that subsequently release downstream basal ganglia pathways to initiate select motor programs. There is extensive convergence at the level of excitatory inputs to the striatum (roughly 10:1) as compared with downstream pallidal and thalamic nuclei, implying that initial processing for action selection occurs at striatal synaptic connections (Zheng and Wilson, 2002; Yim et al., 2011). Despite the large portion of striatal volume covered by many cortico-striatal axons, adjacent MSNs seem to sample unique excitatory inputs, thereby creating sparse striatal representations of cortical firing (Kincaid et al., 1998). Lateral inhibition from recurrent MSN collaterals and feed-forward inhibition from inhibitory interneurons may play a significant role in shaping the activity of neighboring striatal ensembles encoding alternative behaviors (Gage et al., 2010; Chuhma et al., 2011; Yim et al., 2011). Finally, interactions between downstream basal ganglia circuits may further reinforce the striatal selection process (Gittis et al., 2014).

Another proposed function of basal ganglia circuits, and the dorsal striatum in particular, is to encode short motor programs (so-called “chunking” of action repertoires), which can prevent excessive computational demands on cortical structures (Graybiel, 1998). These short motor programs can then be linked together in the dorsal striatum to increase the complexity of motor output (Yin, 2010). When functioning properly, the aforementioned systems should permit efficient selection and assembly of motor programs. However, when dysfunctional, these same networks may be prone to driving the repetitious, automated behavioral patterns frequently observed in ASD. Despite an array of documented striatal morphological abnormalities (see following Section Clinical Indications for Striatal Involvement in ASD), human imaging studies can only suggest a correlation between restricted, repetitive motor output and striatal changes. A small supporting body of evidence comes from two structural magnetic resonance imaging (MRI) studies—the first highlighted a correlation between growth of the caudate nucleus and repetitive behaviors (specifically “resistance to change”) in a longitudinal study of preschool-age children (Langen et al., 2014) and another correlating caudate and putamen volumes with global repetitive behavior metrics (Hollander et al., 2005). Further, research will be needed to assess whether other autism-relevant repetitive symptoms, including motor stereotypies or speech abnormalities, are associated with alterations in basal ganglia morphology.

### Reward-guided behaviors

The midbrain dopamine system has developed together with the sensorimotor circuitry of the basal ganglia to dramatically enhance the manner in which rewards bias an animal's behavior (Murray et al., 2011). Dopamine signaling can increase behavioral efficiency both through its actions on sensory-selection mechanisms (detecting pertinent cues; Berridge, 2007) and action-selection mechanisms (selecting previously rewarded behaviors; Schultz, 2013), although the specific neuronal mechanisms remain controversial. One hypothesis is that striatal interactions with the dopamine system selectively reinforce associations between an environmental cue, a specific response and an outcome to create an internal representation of an animal's action and its consequences. This template could then be used for guiding adaptive behaviors when contingencies change or as a foundation upon which commonly rewarded activities could become automated (Liljeholm and O'Doherty, 2012; Rueda-Orozco and Robbe, 2015).

In rodents, the striatal systems mediating these functions are thought to be segregated, with the dorsal medial striatum (roughly analogous to the caudate in humans) supporting goal-directed behavioral responding, the dorsal lateral striatum (analogous to the putamen) supporting automated behaviors and the nucleus accumbens mediating motivational states and reward processing (Yin and Knowlton, 2006; Balleine and O'Doherty, 2010; Floresco, 2015). In addition, both the dorsal medial striatum and nucleus accumbens are key neural circuits for maintaining flexible behavioral responding under changing reward contingencies (Kehagia et al., 2010). Each striatal domain receives discrete excitatory projections (Pan et al., 2010) and dopaminergic innervation (Lerner et al., 2015) believed to support its specific processing functions, and abnormal coordination between these domains is believed to underlie behavioral control deficits in several neuropsychiatric diseases, including OCD and substance abuse (Voorn et al., 2004; Pan et al., 2010; Russo et al., 2010; van den Heuvel et al., 2010; Ahmari et al., 2013; Burguière et al., 2015). Imaging studies and psychological testing have documented discrete reward-processing deficits in ASD patients, both for social and monetary rewards (Kohls et al., 2013). These abnormalities may contribute to the widespread deficits in motivation and incentive-based learning that are observed clinically (Kohls et al., 2012). Furthermore, a range of deficits in executive function have been observed in high-functioning autistic patients, including alterations in response inhibition, planning, and behavioral flexibility (Pennington and Ozonoff, 1996; Geurts et al., 2004; Hill, 2004; Shafritz et al., 2008). In contrast, other striatal-based paradigms, such as the acquisition of basic operant performance and the ability to coordinate goal-directed and habitual behavioral control seem unchanged (Geurts and de Wit, 2014). Taken together, it seems likely that the profound deficits in social approach and rigid behavioral patterns that typify ASD may stem in part from specific abnormalities in striatal-based reward processing.

### The creation and modulation of behavioral variability

One final consideration with particular relevance to ASD symptomatology is the proposed function of the striatum as a generator of behavioral variation. A wealth of information on the development and context-dependent modulation of highly stereotyped motor output has come from work on a dedicated cortico-basal ganglia circuit that regulates bird-song variability, the anterior forebrain pathway (Fee and Goldberg, 2011). During “practice singing” in isolation, a male's exploration of different song renditions is mediated by variable basal ganglia firing downstream of synchronized striatal output (Woolley et al., 2014). In contrast, song directed at potential female mates is precise—a byproduct of a more stereotyped basal ganglia firing pattern, which may result from cue-dependent increased dopamine release within striatum (Gale and Perkel, 2005; Leblois et al., 2010). While it is currently unclear if the mammalian striatum is similarly involved in the regulation of behavioral variability, it is easy to see how deficits in this function could contribute to the restricted behavioral output observed in ASD.

## Clinical indications for striatal involvement in ASD

Early clinical evidence for striatal involvement in ASD came from widespread “disturbances of motility” noted in neurological testing (Damasio and Maurer, 1978; Maurer and Damasio, 1982). Autistic patients displayed classical neurologic signs of basal ganglia dysfunction including dystonia of the extremities and “striatal toes”—a Babinski-like spontaneous reflex. In addition to involuntary choreoathetoid movements and postural changes, bradykinetic abnormalities were also common, resulting in significant delays in the initiation, modulation and halting of motor output (Maurer and Damasio, 1982). In a cohort of 154 children with ASD from ages 2 through 7, the prevalence of motor abnormalities was substantial, with 51% exhibiting dystonia and 34% motor apraxia (Ming et al., 2007). Magnetic resonance imaging (MRI) studies exploring disease-related changes in striatal volume found evidence for alterations in caudate size both in children and adult ASD patients (Sears et al., 1999; Langen et al., 2007, 2009; Estes et al., 2011). An unbiased meta-analysis of voxel-based morphometric studies taken from the current autism literature has similarly highlighted the basal ganglia as a brain region with consistent structural alteration (Nickl-Jockschat et al., 2012). Alternative approaches have employed functional-MRI to investigate task-specific patterns of striatal activity in ASD patients and uncovered decreased responsiveness during paradigms assaying social reward processing (Delmonte et al., 2012; Kohls et al., 2013) and cognitive flexibility (Shafritz et al., 2008). With regard to the functional connectivity of ASD brains, there is evidence suggesting an increased connectivity between the caudate nucleus and a range of autism-relevant cortical areas, including prefrontal, premotor and parietal areas, observed both in resting-state and task-specific paradigms (Turner et al., 2006; Di Martino et al., 2011).

The abundance of clinical and imaging evidence, when considered together with the diversity of striatal function, presents a compelling argument for a central role of striatal circuits in ASD pathophysiology (see Table 1 for summary). Nonetheless, our understanding of pathophysiological mechanisms ultimately relies on the ability to manipulate systems to test causality. The modeling of ASDs in rodents has largely been pursued through environmental and genetic models, with a focus on construct validity (the disease relevance of how a model was generated) and face validity (how the model recapitulates disease behaviors and pathology; Nestler and Hyman, 2010). Environmental models have been essential in the generation of experimental ASD rodents while the discovery of causal genetic factors was still on the horizon. For example, both prenatal exposure to valproic acid and models of maternal infection can cause social and motor phenotypes that are consistent with abnormalities seen in ASD patients (Arndt et al., 2005). However, our limited understanding of environmental contributions to ASD pathogenesis has severely limited the construct validity of these approaches (McOmish et al., 2014). Genetic modeling in mice has provided alternative disease models whose construct validity rests largely on the quality of genetic association data that serves as the starting point for functional analysis.

**Table 1:** **Brief summary of clinical evidence for the involvement of striatal circuits in ASD pathophysiology**.

**References**	**Population**	**Methodology**	**Conclusion**
Damasio and Maurer, 1978; Maurer and Damasio, 1982	unclear	Neurologic assessment	Classical neurologic signs of basal ganglia dysfunction, including “striatal toes,” choreoathetoid movements, postural changes, and bradykinesia
Sears et al., 1999	12-29 YO	Structural MRI	Enlargement of caudate observed in autism patients; caudate volume associated with compulsions and rituals
Geurts et al., 2004	6-12 YO	Cognitive testing	Children with high-functioning autism exhibited deficits across multiple executive function domains
Turner et al., 2006	15-39 YO males	Functional connectivity MRI	Autism cases displayed decreased functional connectivity in caudate circuits, despite diffusely enhanced connectivity in pericentral regions
Langen et al., 2007	Children and adolescents	Volumetric MRI	Caudate enlargement observed in medication-naïve subjects
Langen et al., 2007	6-25 YO	Structural MRI	Caudate increased in volume with age in autism
Shafritz et al., 2008	Young adult	fMRI	High-functioning autistics had reduced activation in frontal, striatal and parietal regions, as well as lower accuracy on response-shift trials
Estes et al., 2011	3-4 YO	MRI	Enlargement of left and right putamen, left caudate observed in ASD cases
Di Martino et al., 2011	7-13 YO	Functional connectivity MRI	Children with ASD exhibited enhanced striatal connectivity with heteromodal associative and limbic cortices
Delmonte et al., 2012	Teenage males	fMRI	ASD cases showed reduced activation in the dorsal striatum during the receipt of social rewards but normal activation for the receipt of monetary rewards
Nickl-Jockschat et al., 2012	Meta-analysis	MRI	Unbiased meta-analysis of brain structure changes across multiple MRI studies using voxel-based morphometry shows that basal ganglia is significantly affected
Kohls et al., 2013	Teenage males	fMRI	NAc hypo-activation for monetary but not social reward; Amygdala and anterior cingulate cortex hypoactivation for both types of reward
Langen et al., 2014	Preschool children	Structural MRI	Correlation between growth of caudate nucleus and repetitive behaviors (measured as “resistance to change”)

## Striatal dysfunction in mouse genetic models of ASD

**Figure 3:**
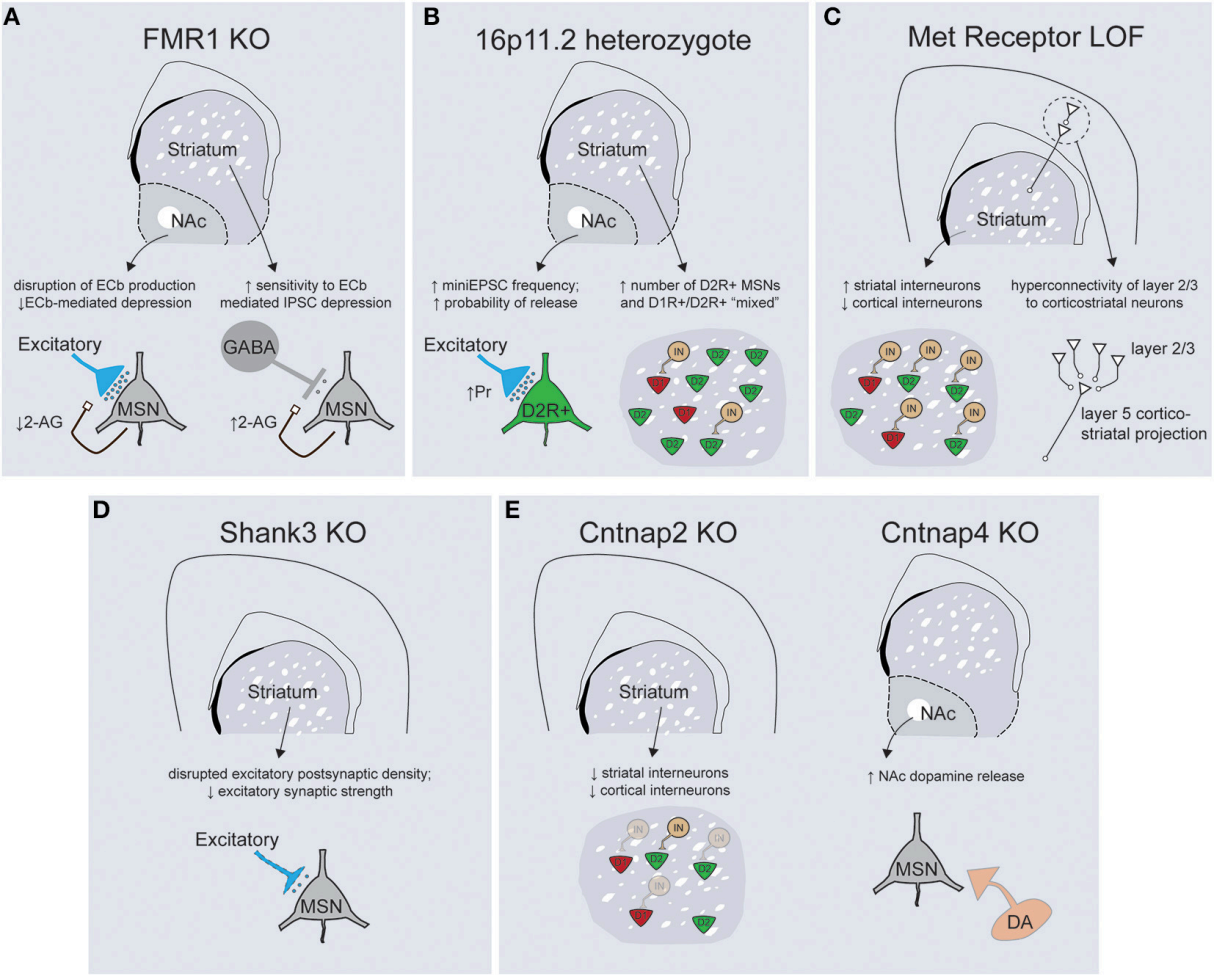
**Mouse models of ASD-associated genes display abnormalities in striatal structure and function. (A)** FMR1 KO mice display abnormalities in the regulation of endocannabinoid signaling within the striatum, with decreased 2-arachidonylglycerol (2-AG) production in the NAc leading to altered plasticity of excitatory inputs (left) and increased 2-AG production in the dorsal striatum enhancing depression of inhibitory transmission onto dorsal striatal MSNs (right). **(B)** 16p11.2 heterozygotes exhibit an increase in excitatory synaptic transmission onto D2R+ MSNs of the NAc (left) while both the dorsal and ventral striatum have larger overall numbers of D2R+ MSNs (right). **(C)** Loss of Met receptor function within ventral telencephalic progenitors leads to an increase in the number of parvalbumin and somatostatin-positive striatal interneurons at the expense of cortical interneuron populations (left). Met receptor KO mice display enhanced connectivity of superficial layer cortical neurons that synapse on corticostriatal projection neurons (right). **(D)** Shank3 KO mice exhibit gross abnormalities in synaptic structure, alterations in the protein architecture of synapses and a decrease in general excitatory synaptic strength within the dorsal striatum. (**E**, left) Cntnap2 KO mice exhibit deficits in migration of interneuron progenitors such that striatal interneuron populations are decreased. Cntnap4 KO mice demonstrate enhanced release of dopamine specifically within the nucleus accumbens (right).

The parallel revolutions in genetic sequencing technologies and genome editing have provided an unparalleled opportunity to explore causality between mutations and aberrant behaviors in genetically tractable systems such as mice. Examinations of copy number variations (CNVs), together with whole-exome and genome sequencing, has demonstrated a substantial amount of genetic heterogeneity underlying ASD etiology, with some estimates predicting that 300–800 loci will eventually be associated with increased risk for ASD (O'Roak et al., 2012). The diversity of documented ASD-associated mutations includes syndromic mutations, rare alleles of larger effect size, de-novo CNVs and more common mutant alleles of smaller phenotypic penetrance (Krumm et al., 2014; Willsey and State, 2015). Genetic modeling of ASD in mice has largely focused on syndromic mutations and rare alleles, although the generation of CNV models has recently gained traction (Nakatani et al., 2009; Horev et al., 2011; Portmann et al., 2014). My goal here is not to exhaustively review each mouse ASD model, but instead provide an overview of the physiological and behavioral phenotypes resulting from mutations in a range of genes with solid genetic association to ASD. When considered together, I believe these data begin to make a strong case for primary striatal deficits in the pathogenesis of ASD (Figure 3).

### Fragile-X mental retardation protein

The Fragile-X Mental Retardation Protein (FMRP), a key repressor of translation at central synapses, has been a central model for exploring the pathogenesis of mental retardation and accompanying developmental disorders including autism (Danish-Belgium Fragile-X Consortium, 1994; Reiss et al., 1995; Bear et al., 2004). While this protein exerts widespread control of mRNA translation, many of the neuropsychiatric-related symptoms seen in Fmr1 KO mice result from abnormal activation of the mGluR5 metabotropic glutamate receptor, as evidenced by the striking behavioral rescue achieved in Fmr1 KO; mGluR5 heterozygote mice (Dölen et al., 2007). While initial studies described a role for Fmr1 in regulating long-term synaptic plasticity at hippocampal synapses (Huber et al., 2002), it has subsequently been implicated in synaptic dysfunction across multiple brain regions, including cingulate cortex, amygdala, and neocortex (Patel et al., 2013; Martin et al., 2014; Koga et al., 2015). The idea that fronto-striatal circuit dysfunction is critical to specific domains of behavioral dysfunction seen in Fragile X patients is suggested by deficits of response inhibition and abnormal patterns of task-related activity in anterior cingulate cortex and striatum (Menon et al., 2004). Interestingly, this study also noted that responses in the ventrolateral prefrontal cortex and the striatum were correlated with the levels of Fmr1 gene expression. The importance of fronto-striatal dysfunction has received additional support from recent cognitive studies in the Fmr1 mouse model demonstrating abnormalities in visuospatial discrimination and extinction of instrumental responses (Krueger et al., 2011; Sidorov et al., 2014). Consistent with the importance of fronto-striatal circuits, synaptic analysis of Fmr1 KO mice has demonstrated alterations in both excitatory and inhibitory synaptic transmission within the ventral and dorsal striatum, respectively (Figure 3A; Centonze et al., 2008; Jung et al., 2012). In the dorsal striatum Fmr1 KOs display an increased sensitivity to endocannabinoid-mediated depression of inhibitory transmission onto MSNs, while in the nucleus accumbens a form of endocannabinoid-mediated depression of excitatory transmission is disrupted (Centonze et al., 2008; Jung et al., 2012). These studies illustrate how a common mutation may yield similar net circuit effects (less inhibition of striatal MSNs) through region-specific physiological mechanisms. Nevertheless, the widespread nature of physiological dysfunction that occurs downstream of perturbations to this global regulator of translation makes it currently unclear what specific role striatal dysfunction might play in the diverse behavioral changes documented in Fmr1 KO mice.

### Mouse models of the 16p11.2 human CNV

Copy number variations on chromosome 16p11.2 are one of the most common sequence abnormalities associated with ASD (Weiss et al., 2008). Deletions of this region cause a range of phenotypes in addition to autism, including language delay, seizures, cognitive impairments and attention-deficit hyperactivity disorder, while duplications are associated with schizophrenia. The 16p11.2 chromosomal region contains 26 genes whose orientation in humans is perfectly conserved on chromosome 7 in mice, allowing faithful genetic modeling (Horev et al., 2011; Portmann et al., 2014). Consistent with the large size of genetic insult, 16p11.2 heterozygote mice displayed gross abnormalities in brain morphology and size. In particular, rostral striatum, nucleus accumbens, globus pallidus, medial cortical structures, and thalamus all exhibited enlargement, suggestive of a coordinated increase in the morphological footprint of basal ganglia circuitry. Detailed anatomical analysis revealed evidence for changes in medium spiny neuron specification, with increased overall numbers of D2R+ MSNs as well as a larger fraction of spiny neurons with “mixed” D1R+ and D2R+ phenotypes. Furthermore, there was a dramatic increase in the net excitatory strength onto D2R+ MSNs secondary to a cell-type specific increase in the presynaptic probability of neurotransmitter release (Portmann et al., 2014). All together, these changes should act to enhance output from the indirect pathway in both the dorsal and ventral striatum, although the resulting changes at the circuit level should be interpreted with caution in the face of what seems to be significant abnormalities in neural specification and development (Figure 3B). Further work is necessary to conclude whether these striatal circuit alterations play a causal role in the numerous motor behaviors documented by this study, as 16p11.2 deletions have also been associated with cortical dysplasia and aberrant hippocampal mGluR signaling (Pucilowska et al., 2015; Tian et al., 2015).

### Met receptor signaling

The Met receptor tyrosine kinase binds its ligand, hepatocyte growth factor, and mediates numerous signaling events essential for development of epithelial populations including proliferation, differentiation, and trophic support. In the developing nervous system, Met is expressed in proliferative progenitor zones and maintained during neuronal migration and integration, suggesting Met signaling is essential for early neuronal specification (Powell et al., 2001). Consistent with this, mutations in the Met receptor have been associated with both autism and Tourette's syndrome (Martins et al., 2011; Peng et al., 2013). The role that Met dysfunction plays in the pathophysiology of these disorders is unclear, but targeted genetic dissection has demonstrated abnormalities at multiple levels of cortico-striatal circuitry (Figure 3C). Using two-photon glutamate uncaging to explore circuit-specific synaptic connectivity, it was shown that cortical disruption of Met function caused hyper connectivity of cortical layer 2/3 neurons specifically onto layer 5 cortical neurons that projected into the striatum (Qiu et al., 2011). Cortical-specific Met disruption also resulted in a non-cell autonomous increase in the total dendritic arbor length and spine volume of striatal MSNs (Smith et al., 2012). An alternative approach that removed Met signaling from ventral neural progenitors produced abnormalities in the proper distribution of inhibitory interneurons, such that the number of parvalbumin- and somatostatin-positive interneuron subtypes was increased in the striatum at the expense of cortical populations (Martins et al., 2011). It is difficult to estimate what the net circuit effect of these seemingly opposing alterations might be—while cortical Met disruption should enhance cortico-striatal network output, too little is known about the putative functions of striatal interneurons to extrapolate the result of enhanced local inhibition on overall striatal processing (Gage et al., 2010; Yim et al., 2011). Nonetheless, further work is necessary to directly attribute alterations in corticostriatal pathways to the deficits in procedural and reversal learning seen with Met genetic loss-of-function (Martins et al., 2011).

### The shank gene family

Shank proteins are a central component of the postsynaptic density that function to scaffold the large protein networks associated with excitatory synapses. While all three Shank family members are associated with ASD, the strongest genetic evidence exists for Shank-3, whose location maps to the critical region for Phelan-McDermid syndrome—a disorder characterized by intellectual disability, autistic behaviors and hypotonia (Phelan, 2008). Understanding how Shank-3 mutations cause neuropsychiatric disease has been complicated by the molecule's complex structure, which employs multiple transcriptional start sites to generate proteins with numerous adhesion domains (Jiang and Ehlers, 2013). Genetic alterations have been detected throughout the Shank-3 gene and the variability of resulting phenotypes provides insight into the relative importance of the specific adhesion domains that are disrupted. For example, mice in which the upstream ankyrin repeats are affected have specific reductions in the Shank3α protein isoform and relatively mild behavioral phenotypes compared to mutations in the downstream PDZ domain, which eliminate the α, β, and γ isoforms and produce significant increases in grooming behaviors and reductions in social interactions (Peca et al., 2011). Biochemical and physiological experiments focused on striatum because of the severe grooming phenotype and the high levels of Shank-3 mRNA expression selectively within this structure. These analyses demonstrated truncated postsynaptic density structure along with broad reductions in key scaffolding proteins and neurotransmitter receptors (Peca et al., 2011). Consistent with this, Shank-3 KOs have large reductions in cortico-striatal excitatory synaptic transmission, although whether there is cell-type or input specificity to this deficit remains unclear (Figure 3D). Despite the smaller number of Shank-2 mutations associated with ASD, two distinct mouse models of Shank-2 display social deficits and abnormalities of motor output despite exhibiting opposite hippocampal synaptic phenotypes (Schmeisser et al., 2012; Won et al., 2012).

### Contactin associated proteins

Contactin associated proteins are transmembrane molecules of the neurexin superfamily that have been linked to ASD and epilepsy through numerous human genetic approaches (Strauss et al., 2006; Alarcón et al., 2008; Arking et al., 2008; Bakkaloglu et al., 2008). Contactin associated protein-like 2 (Cntnap2) is implicated in neuron-glial interactions and clustering of potassium channels at the nodes of myelinated axons (Poliak et al., 1999). Recent work in cultured cortical neurons has also demonstrated a potential developmental role in the elaboration of dendritic arbors and development of synaptic spines (Anderson et al., 2012). Mouse models of Cntnap2 loss-of-function display stereotypic movements, behavioral inflexibility, social and communication deficits, as well as seizures (Peñagarikano et al., 2011). While the underlying neural mechanisms of these behaviors remain unclear, there are widespread abnormalities in the migration of inhibitory interneurons, leading to a decrease in both cortical and striatal interneuron populations (Figure 3E; Peñagarikano et al., 2011). Given the phenotypes of the Met receptor (Martins et al., 2011) and Cntnap2 KOs, further studies are needed to explore whether alterations in striatal interneuron development represent a common causal factor for ASD pathogenesis. Cntnap4 is a closely related family member that has been associated with neuropsychiatric disease whose expression is restricted to parvalbumin-positive interneurons and tyrosine hydroxylase positive midbrain dopamine neurons in the substantia nigra pars compacta and ventral tegmental area (Karayannis et al., 2014). Cntnap4 protein is expressed presynaptically and KO mice display diverse synaptic phenotypes including a reduction in cortical GABAergic tone and an increase in release of dopamine specifically within the nucleus accumbens (Figure 3E). The perseverative grooming displayed by Cntnap4 KOs is lessened by systemic administration of the dopamine D2 receptor antagonist haloperidol, suggesting that increased dopaminergic tone is in part responsible for the observed motor control abnormalities (Karayannis et al., 2014). Taken together, functional data from mutations in the Cntnap family suggest widespread abnormalities in inhibitory function and focal changes in local dopaminergic release. Whether, these changes converge at the level of striatal circuits is an interesting future question.

## Directly exploring the link between gene dysfunction, circuit abnormalities and ASD-relevant behaviors

**Figure 4:**
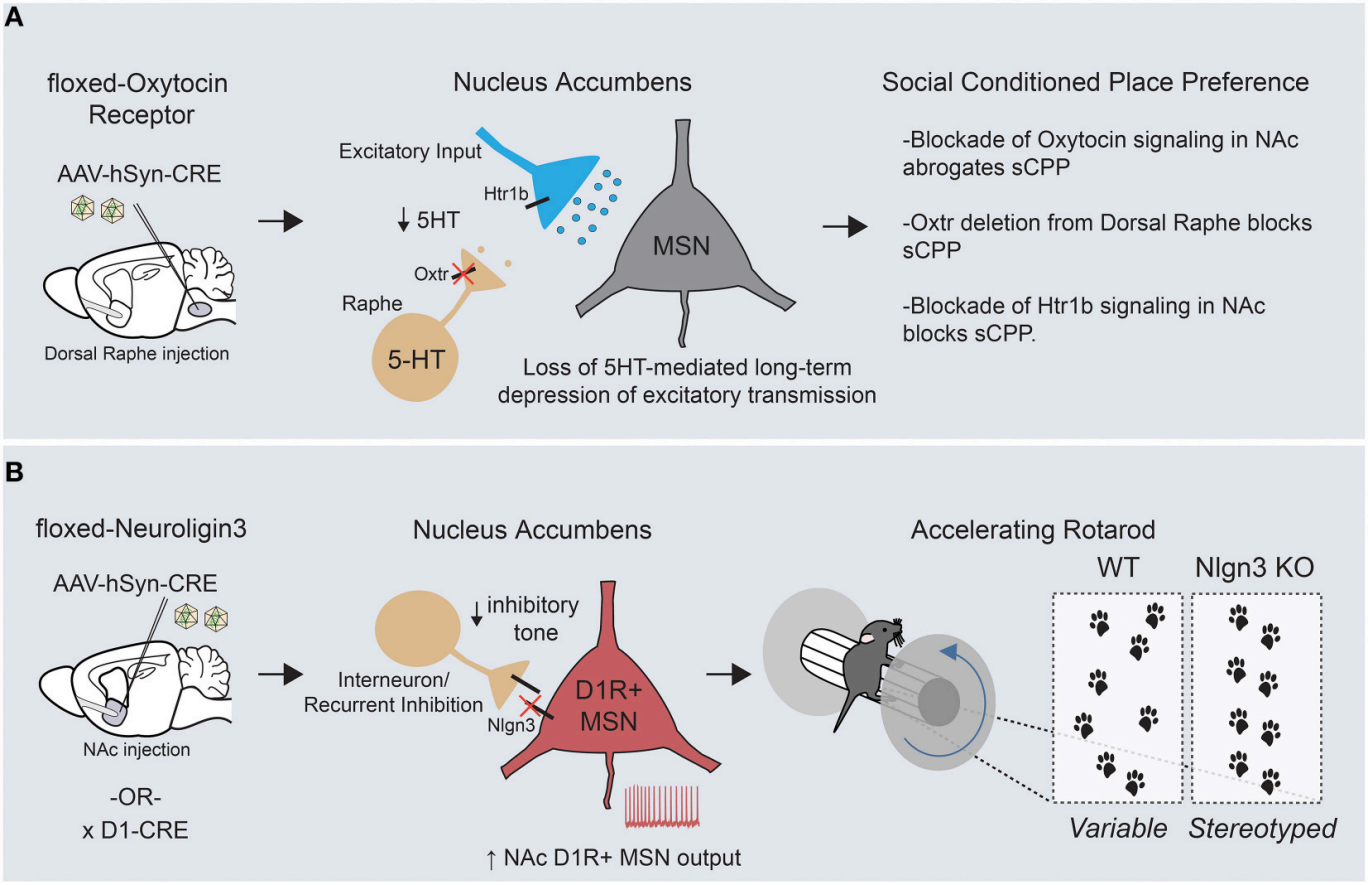
**Circuit-specific analysis of ASD-related behaviors points toward an underlying striatal deficit. (A)** Targeted removal of oxytocin receptor within the dorsal raphe demonstrates a key role for this molecule in formation of social conditioned place preference, a measure of social reward. Oxytocin receptor functions on dorsal raphe terminals within the nucleus accumbens to regulate the release of serotonin, which can hetero-synaptically modulate excitatory transmission onto MSNs through presynaptic Htr1b receptors. **(B)** Removal of Neuroligin-3, a synaptic adhesion molecule associated with ASD, from D1R+ MSNs of the nucleus accumbens is sufficient to drive the enhanced formation of repetitive motor routines, as assayed by learning on the accelerating rotarod (KOs display an increased and earlier stereotyped pattern of foot placements compared with WTs). In addition, Neuroligin-3 KO mice have a cell type- specific deficit in inhibitory transmission onto accumbens D1R+ MSNs, which presumably leads to an increase in output from this circuit.

Taken together, these studies demonstrate that the introduction of ASD-associated mutations into mice causes dysfunction of striatal structure and function. However, an equally compelling case could be made for ASD-associated abnormalities in hippocampal CA1 neurons, a cell type in which many autism-associated mutations have been screened due to their well-characterized connectivity and anatomy (Etherton et al., 2011; Peñagarikano et al., 2011). In fact, most of the aforementioned genes produce physiological changes in multiple brain regions. To further our understanding of ASD pathophysiology, it is necessary to move beyond the basic concept that ASD-associated genes cause physiological abnormalities to instead explore which behaviorally relevant neural circuits are changed and how those networks function to normally regulate behavior (Fuccillo et al., 2016). Two recent studies have tackled this issue by employing viral and genetic dissection of gene function toward the goal of a circuit-based understanding of ASD-relevant behavioral abnormalities (Figure 4; discussed in Section Striatal Oxytocin Function in Social Reward and Striatal Neuroligin-3 Dysfunction Boosts Repetitive Behaviors; Dölen et al., 2013; Rothwell et al., 2014).

### Striatal oxytocin function in social reward

Abnormalities in reward processing likely contribute to the widespread deficits in social engagement and communication seen in ASD patients. In support of this, human imaging studies have demonstrated reduced neural activity throughout corticostriatal circuits in response to a variety of social rewards (Kohls et al., 2012). To better understand the circuit mechanisms of social behaviors, a conditioned place preference assay (similar to that used to examine the rewarding properties of drugs of abuse) was employed to quantify a preference for contexts associated with grouped vs. isolation housing (Panksepp and Lahvis, 2007; Dölen et al., 2013). Using this approach, the function of oxytocin, an ancestral neuropeptide known to regulate affiliative behavior across many species, was mechanistically examined. Alterations in the oxytocin promoter have been associated with ASD and intra-nasal oxytocin delivery is currently being explored as a treatment option to enhance pro-social behaviors (Yamasue et al., 2012). Through a combination of acute slice electrophysiology and behavioral pharmacology, two interesting but seemingly disconnected effects of oxytocin were demonstrated in mice—(1) bath application of oxytocin induced a long lasting depression of excitatory synaptic transmission onto MSNs of the nucleus accumbens, and (2) blockade of oxytocin signaling in the NAc inhibited the formation of a preference for social cues (Dölen et al., 2013). Viral-mediated circuit dissection of oxytocin receptor (Oxtr) function demonstrated that both the synaptic plasticity and the social preference behavior depended on oxytocin-ergic signaling in dorsal raphe inputs to the NAc, a major source of striatal serotonin.

Through a series of experiments, a unifying mechanism emerged in which oxytocin signaling regulated the release of serotonin in the nucleus accumbens, which subsequently modulated excitatory synaptic strength by acting at presynaptic serotonergic receptors on excitatory afferent fibers (Figure 4A). Despite the complexity of interactions across several brain regions, it is clear that the nucleus accumbens is the final locus at which this modulation of social reward occurs, as both oxytocin and serotonin-1B (Htr1b) receptor blockade in the accumbens abrogate the social preference *in vivo*. Questions remain regarding how these long-term changes in accumbal excitatory transmission ultimately regulate social reward. Nonetheless, these experiments have made the first inroads into understanding the synaptic basis of social reward processing and furthermore offer potential mechanistic clues to the function of two ASD-associated genes (Oxtr and Htr1b) in the development of ASD-associated behaviors.

### Striatal neuroligin-3 dysfunction boosts repetitive behaviors

Applying a similar “circuit dissection” approach, we attempted to explore how ASD-associated mutations could promote development of the restricted and repetitive behaviors so frequently observed in ASD patients (Rothwell et al., 2014). To do so, we employed the accelerating rotating rod (rotarod) as a potential behavioral endophenotype for the formation of motor routines. To assess the validity of this approach, we used video-capture foot tracking of mice during standard rotarod training to demonstrate that improved motor performance was tightly linked to increasingly stereotyped location and timing of hind-paw placement. We then investigated this behavior in two distinct mouse lines mutant for Neuroligin-3, a synaptic adhesion molecule associated with ASD (Jamain et al., 2003; Sanders et al., 2011). Interestingly, both Neuroligin-3 KO and Neuroligin-3 R451C point mutant mice demonstrated enhanced learning on the rotating rod and a more rapid stereotyping of their paw placement. This finding is of great significance given that several genetic ASD models have a similar enhancement of rotarod performance (Kwon et al., 2006; Nakatani et al., 2009; Etherton et al., 2011; Peñagarikano et al., 2011). Using a series of viral and genetic approaches, Neuroligin-3 was removed from discrete circuits and cell types in an attempt to delineate where Neuroligin-3 dysfunction was crucial for enhancing rotorod learning and other stereotyped behaviors. Surprisingly, our data suggested that deletion of Neuroligin-3 in D1R+ MSNs of the mature nucleus accumbens was sufficient to generate the behavioral phenotypes seen in two separate Neuroligin-3 autism models. Complementary acute slice analyses demonstrated a deficit in inhibitory synaptic transmission specifically onto D1R+ MSNs of the accumbens, which altered the neuronal balance between excitation and inhibition in this circuit element (Figure 4B).

To prove that altered nucleus accumbens MSN output can modulate rotarod acquisition, we employed cell type-specific suppression of neuronal activity through targeted stereotaxic injection of a Cre-dependent virus expressing the inwardly-rectifying potassium channel, Kir2.1. These experiments demonstrated that decreasing excitability of D1R+ and D2R+ MSNs could bi-directionally modulate rotarod learning, and strongly suggests that the synaptic dis-inhibition of accumbal D1R+ MSNs is a causal physiological event in the promotion of repetitive motor behaviors in Neuroligin-3 mutant mice. How exactly changes in nucleus accumbens D1R+ MSN output help to organize motor patterns is intriguing given that these programs are likely executed elsewhere within cortico-striatal circuits. Along these lines, it is important to note the critical differences in downstream connectivity recently reported between medium spiny neuron subtypes of the dorsal vs. ventral striatum (Kupchik et al., 2015; see Figure 1C). Whereas D1R+ MSNs of the dorsal striatum project exclusively through the direct pathway to the midbrain, D1R+ MSNs of the nucleus accumbens send inhibitory projections to both mesencephalic dopamine centers and the ventral pallidum, the main target site of D2R+ accumbal MSNs. This distinction suggests that there are two possible downstream pathways through which alterations in D1R+ MSN output could shape repetitive behaviors. Together with the aforementioned oxytocin study, these results begin to show a clustering of causal ASD-related neural dysfunction within the striatum. Further work will be necessary on both fronts to better understand how these circuit abnormalities interact with global brain function to alter behavior.

## Can understanding striatal circuit regulation of behavior help generate hypotheses of ASD pathophysiology?

Given the recent evidence that striatal dysfunction has a causal role in ASD-related behaviors, a more comprehensive understanding of how specific striatal circuits mediate behavioral control may aid our attempts to forge mutation-behavior correlates in rodent disease models. Lesion studies, pharmacological manipulations and more recently, optogenetic/pharmacogenetic interrogation of striatal circuits has begun to create a cellular and synaptic understanding for the selection and reinforcement of particular behavioral patterns.

### Striatal regulation of flexible and automatic behavioral responses

Striatal function is believed to support both flexible, goal-directed behaviors as well as more automated responding, in an attempt to enhance overall behavioral efficiency. How these two systems interact to shape behavior is of considerable importance given their widespread dysfunction across multiple neuropsychiatric disorders. Lesion studies in rodents have attributed goal-directed responding to dorsomedial striatal function, as disruptions of this territory generate rigid behavioral patterns that are insensitive to reward devaluation (Yin et al., 2005). Furthermore, acquisition of goal-directed actions is correlated with bidirectional synaptic plasticity within the dorsomedial striatum, with enhanced AMPA receptor mediated synaptic transmission onto D1R+ MSNs and decreased excitatory synaptic drive onto D2R+ MSNs (Shan et al., 2014). These data reinforce a basic circuit logic whereby enhanced drive onto D1R+ MSNs serves to boost selected behaviors while decreased activation of D2R+ MSNs allows for the removal of an inhibitory brake. Finally, dorsomedial striatal MSNs also seem to have a role in encoding the net expected return of a given task, and modulating response vigor accordingly (Wang et al., 2013). This regulation of task effort may prove integral to the function of the dorsomedial striatum in shaping aspects of reward-sensitive associative learning.

Lesion studies of the rodent dorsolateral striatum suggest that this domain is necessary for the formation of habits—automated, sensory-driven responses that are insensitive to changes in reward value or contingency (Yin et al., 2004; Yin and Knowlton, 2006). In addition, inactivation of the dorsolateral striatum after the establishment of habitual responding causes a reversion to more reward-sensitive behavioral output, suggesting either parallel or antagonistic interactions between these two systems. The neural mechanisms that coordinate reward-directed flexibility and fixed motor responding have received little attention but are likely of significant importance to ASD pathology. Unfortunately, a strict anatomical segregation of these two processes is unlikely, as evidenced by state-related MSN activity in both dorsomedial and dorsolateral striatal compartments during goal-directed and habitual responding (Gremel and Costa, 2013).

### Intrinsic striatal circuits and goal-directed learning

Traditional models of striatal function are grounded in the proposed dichotomy of striatal medium spiny neuron subtypes, with D1R+ MSN activity important for initiating movement and D2R+ MSNs essential for suppressing actions. While the precise temporal sequence and coordination of MSN subtypes during activity remains unclear (Cui et al., 2013), this theoretical framework has been a powerful tool for progress in understanding striatal motor function (Kravitz et al., 2010). Recent work has attempted to explore whether these striatal cell types have analogous functions in the regulation of reinforced actions. Mice expressing channelrhodopsin in either direct or indirect MSNs of the dorsomedial striatum were allowed to lever press to receive optogenetic activation of either circuit component (Kravitz et al., 2012). In this optogenetic variant of self-stimulation, it was noted that stimulation of D1R+ MSNs resulted in persistent operant reinforcement that was maintained across training session, while D2R+ MSN stimulation caused a transient aversive state within each session. Surprisingly, both cell-type specific modulations of behavior occurred in the presence of systemic dopamine antagonists, suggesting that the behavioral plasticity was occurring through alternative mechanisms. Another study focused specifically on direct pathway neurons of the dorsomedial striatum, employing DREADDs technology to increase or decrease G-protein coupled signaling in this cell type during the acquisition of a high vs. low reward-discrimination task (Ferguson et al., 2013). While these manipulations had no real-time effect on reward preference or task acquisition, they were able to bi-directionally modulate the encoding of strategies for subsequent trials. A comparison between these optogenetic and pharmocogenetic manipulations of MSN activity demonstrates how distinct temporal windows of activity within the same neural circuit can mediate diverse aspects of behavioral control.

Similar cell type-specific functions have been proposed for MSN cell types within the nucleus accumbens, as demonstrated by a cell type-specific block of synaptic transmission through regulated viral expression of tetanus toxin (Hikida et al., 2010). Using locomotor sensitization and conditioned-place preference behaviors to explore MSN contributions to psycho-stimulant exposure, the authors suggest that D1R+ MSNs mediate a broad set of associative reward functions. In contrast, D2R+ MSNs were essential for the aversive response to foot-shocks seen in the inhibitory avoidance task. A separate study exploring the transition to compulsive cocaine use during self-administration paradigms suggests that increased synaptic strength onto D2R+ MSNs of the nucleus accumbens was associated with resilience to chronic cocaine use (Bock et al., 2013). These results argue that the indirect pathway may shape behaviors by curtailing unwanted or maladaptive efforts. In fact, the interpretation that D2R+ MSN activity encodes aversive states may reflect this cell type's function in suppression of action associated with non-rewarded or aversive contexts. When taken together, the data thus far suggest that D1R+ MSNs in the NAc and associative striatum have a key role in goal-directed actions and learning, while D2R+ MSNs in these regions may encode either aversive states or mediate the inhibition of specific behaviors associated with these states.

### Striatal circuit modulation and flexible behavioral responding

Current theories of striatal function highlight the importance of afferent projection neurons and neuromodulatory populations in controlling the final output of basal ganglia circuits, however few studies have functionally tested these assumptions (Mathur and Lovinger, 2012; Tritsch and Sabatini, 2012; Wall et al., 2013; Guo et al., 2015). Recent lesion experiments have demonstrated how the central nucleus of the amygdala (CeA) may interact with the dorsolateral striatum to regulate the balance between goal-directed and habitual responding (Lingawi and Balleine, 2012). Rats with asymmetrical lesions of the CeA and dorsolateral striatum demonstrated an inability to form habitual responding (defined as becoming insensitive to reward devaluation), despite extensive training. How communication between these regions regulates a switch to habitual responding is unclear, although other amygdalar regions such as the basolateral nucleus have been shown to gate the plasticity of cortico-striatal synapses through NMDA-dependent mechanisms (Popescu et al., 2007). Another fascinating demonstration of striatal afferent-mediated regulation of behavioral control comes from attempts to use optogenetics to ameliorate compulsive grooming behaviors seen with deletion of Sapap-3, a synaptic scaffolding gene (Burguière et al., 2013). These mutant mice display behavioral control abnormalities in a cued-grooming task that likely results from dysfunctional inhibition of MSN activity. Surprisingly, this impulse-control deficit could be improved by optogenetic activation of excitatory projections from the lateral orbitofrontal cortex to the dorsal striatum. Increased recruitment of this circuit was able to compensate for striatal inhibitory deficits and restore MSN inhibition, suggesting that cortical regulation of local striatal inhibition is a potentially powerful mechanism for regulating normal behavioral output.

## The search for ASD-relevant circuit dysfunction

The growing catalog of abnormalities—including structural and functional changes observed with imaging of autistic patients and physiological abnormalities documented in mouse genetic models for autism—highlights the importance of discerning commonalities of circuit dysfunction as a path toward understanding disease pathophysiology. While I have focused on striatal dysfunction, a growing body of evidence has accumulated implicating other regions in ASD pathology, including the cerebellum and cortex (Zikopoulos and Barbas, 2010, 2013; Wang et al., 2014). The recurrent phenotypes exhibited by patients and genetic mouse models begs the question as to why particular circuits would be intimately associated with ASD. One possibility is that these circuits have some specific molecular vulnerability rendering them more likely to become dysfunctional in response to a given genetic insult. An alternative hypothesis would be that despite ASD-relevant mutations having widespread effects on physiology, ASD-associated circuits occupy central and convergent points of processing which are uniquely sensitive to neuronal dysfunction and regulate motor and cognitive behaviors typically associated with ASD. Preliminary evidence exists for both of these concepts.

### Do particular circuits exhibit a unique molecular vulnerability?

One can imagine several related mechanisms that would create a circuit-specific vulnerability to genetic insult—(1) neurons either uniquely or more highly express ASD-associated genes such that loss-of-function is more acutely sensed, or (2) neurons do not express alternative family members of ASD-associated genes that would typically allow for genetic compensation. Using a computational approach that analyzed autism genetic datasets to correlate mutations to known biological networks, it was found that autism-associated mutations are preferentially found in genes whose expression levels are enriched in both populations of striatal medium spiny neuron, as well as cortical inhibitory and projection populations, deep cerebellar nuclei and layer 6 corticothalamic neurons (Chang et al., 2015). Another study employing high-confidence autism genes as a “seed” from which to build co-expression networks implicated specific brain region and developmental windows, including mid-fetal cortical development, as well as postnatal thalamic and cerebellar nuclei, in disease pathogenesis (Willsey et al., 2013). Together, these data provide a relatively straightforward explanation for the observed bias toward cortico-striato-thalamic and cerebellar circuit dysfunction seen in ASD patients. In addition to being enriched for ASD-associated genes, it is currently unclear whether lack of molecular redundancy in these circuits also contributes to increased vulnerability to genetic insult. Single neuron transcriptional profiling, with its ability to quantitatively assess mRNA levels across multiple families of ASD-associated genes will be an essential tool in further exploring the molecular vulnerability of neural circuits (Fuccillo et al., 2015).

Beyond possessing a molecular susceptibility for dysfunction, it is interesting to consider whether the evolutionary history of particular neural circuits has contributed to their predisposition for involvement in both ASD and neuropsychiatric disease more broadly. Anatomical and physiological analysis of the basal ganglia in lamprey, the oldest vertebrate lineage, shows a surprising degree of circuit and cell type conservation (Stephenson-Jones et al., 2011). The authors concluded that this reflects a process of “exaptation,” wherein a core ancestral unit is repurposed by natural selection into a structure with altered function (Gould and Vrba, 1982). In this context, the lamprey basal ganglia, which functioned to control basic motor output has been co-opted over time for use by higher vertebrates in the processing of complex cognitive and emotionally driven actions. If true, the evolution of striatal circuits represents a parsimonious solution to enhance the range of behavioral output. However, this constant increase in the complexity of striatal integration may have come at a price. Might more molecules be required in striatal MSNs to integrate these new inputs? Would more spatially precise dendritic targeting of these proteins be required? Does this type of repurposing render the newer striatal functionalities of cognitive and emotional behavioral control less stable than the original motor control circuits? Might this hypothesis hold true for other highly conserved structures such as the cerebellum? Answers to these fascinating questions will require both technical advances and rigorous comparisons between the circuit connectivity of lower vertebrates and their human ancestors.

### Are abnormalities in striatal circuits likely to cause widespread behavioral dysfunction?

An alternative hypothesis for the importance of cortico-striato-thalamic and cerebellar abnormalities in ASD pathophysiology is not that these circuits are uniquely sensitive to genetic mutations, but rather their physiological dysfunction consistently produces robust deficits in social behavior and motor control. As previously discussed, striatal circuits occupy an intersection between internal representations of sensory input, prior experience, motivational state and motor control, and thus are capable of regulating behaviors that are typically linked to ASD. These striatal circuits further serve to regulate the output of the midbrain dopamine system, which extends a widespread neuromodulatory influence throughout the brain (Kupchik et al., 2015; Lerner et al., 2015). Dopamine signaling has been proposed to subserve multiple behavioral functions, depending both on the locus and timescale of its action (Schultz, 2007). Given this, the disruption in striatal function seen in ASD may serve to initiate disparate behavioral changes through brain region-specific dysregulation of dopamine release. In this model, motor control deficits would result from altered dopamine signaling in dorsal striatal structures while abnormalities in reward processing would be secondary to abnormalities in ventral striatal or prefrontal cortical dopamine signaling. Careful mechanistic studies in genetic mouse models will be necessary to further develop these concepts.

### How might cerebellar and cortical circuit dysfunction produce ASD-associated behaviors?

Are there other neural circuits as centrally placed as the striatum for the regulation of behavioral output? Cerebellar and prefrontal cortical circuits also appear as key candidates with influence on both the cognitive and motor aspects of ASD pathology. Given the importance of the cerebellum in motor control and balance, its perhaps unsurprising that the initial discovery of vermal hypoplasia in autistic patients (Courchesne et al., 1988) has been followed by studies linking autism with changes in cerebellar function during basic motor tasks, adaptation of saccades and feedback/feedforward regulation of grasping (Allen et al., 2004; Mosconi et al., 2013, 2015). However, the cerebellum exhibits significant connectivity with cognitive and affective brain regions and its function in language processing and aspects of social cognition may also contribute to autism symptomatology (Reeber et al., 2013; Wang et al., 2014). The extensive connectivity between the cortex and the cerebellum, together with the significant effect of early cerebellar lesions on cognition and social function as opposed to motor control, have led some to hypothesize the existence of a critical period for cerebellar function (Wang et al., 2014). Disruptions during this period may perturb the plasticity and development of cortical regions or block the early-stage learning of basic motor patterns and skills. A related hypothesis posits the cerebellum as an iterative processing unit essential for all motor and cognitive tasks, either through its general regulation of timing or orchestration of widespread neuronal adaptations for skilled motor output (for detailed review, see Strick et al., 2009). Although beyond the scope of this review, it would be interesting to explore the potential for interactions between the cerebellar and striatal systems with regard to autism pathophysiology. Tracing studies have highlighted two interesting points of intersection—(1) the connection of the deep cerebellar nuclei with the dorsal striatum, through a di-synaptic thalamic relay (Ichinohe et al., 2000; Hoshi et al., 2005), and (2) the mono-synaptic projection of deep cerebellar nuclei to the ventral tegmental area (Phillipson, 1979).

Cortical regions, particularly domains within the prefrontal cortex, have also been proposed as sites of dysfunction in ASDs. In rodents, the prefrontal cortex is a heterogeneous anatomical structure comprised of the orbital, cingulate, prelimbic, infralimbic, and agranular cortices (Heidbreder and Groenewegen, 2003). Tracing studies have revealed an extensive connectivity of prefrontal cortex, suggesting it functions to integrate input from sensory, limbic, and autonomic systems (Groenewegen and Uylings, 2000). Prefrontal dysfunction has been proposed to account for abnormalities of social cognition, action control and multi-sensory integration seen in autistic patients (see Martinez-Sanchis, 2014; Bicks et al., 2015; Chmielewski and Beste, 2015 for detailed review). Social cognition and action control both rely upon the integration of motivational states, knowledge of specific environmental or social contexts and the flexible adjustment of behavior. The circuit bases of such wide-ranging functions remains fuzzy and warrant greater study. However, it seems likely that interactions between prefrontal circuits, the striatum and the thalamus will play a key role.

## Future progress in understanding ASD pathophysiology

An integrated picture of ASD pathophysiology will clearly require further research studies that probe, in a non-biased manner, for circuit dysfunctions mediating discrete aspects of ASD symptomatology. Animal models, which allow for direct tests of causality, can provide mechanistic hypotheses that can be fully explored via functional imaging during human psychological testing.

### Making progress with striatal-based models of ASD

Multiple lines of evidence from clinical imaging and rodent disease models have converged to suggest that striatal dysfunction is intimately associated with the etiology and pathophysiology of ASD. This knowledge provides an initial foothold into understanding how genetic abnormalities perturb neuronal and circuit function to generate the complex range of behavioral abnormalities seen in ASD. An important next step will be to assess whether the extreme genetic diversity of ASD-associated genes can be distilled down to a smaller number of circuit or cell type-specific deficits. Comparative physiological and behavioral analyses between pre-existing ASD model mice should aim to discern common behavioral deficits and ascertain whether they are attributable to conserved striatal abnormalities. Elucidation of a recurrent cell type, synaptic or circuit-specific deficit contributing to ASD-related behaviors would dramatically help in focusing future treatment endeavors. This information could then be integrated with the molecular profiles of affected circuit components in search of novel targets for disease amelioration.

Another key step in exploring ASD pathophysiology is to increase the biological relevance of rodent-based disease studies so that their results can be translated and built upon in the pre-clinical setting. To date, a large portion of disease modeling in rodents has focused on mice that are homozygous for functionally null alleles of ASD-associated genes. While this approach has no doubt improved our ability to detect abnormalities, it does so within a biological context that lies far outside the physiological range. The analysis of mutations in a more physiological setting can be achieved through numerous approaches—(1) examining heterozygous mutations, disease-associated point mutations or genetic modifiers, (2) exploration of environmental interactions with mutations (3) physiological analysis of behaviorally relevant circuits through selective optogenetic recruitment and (4) highly quantitative analysis to detect subtle changes in discrete components of complex behaviors. Even with these improvements, it is important to acknowledge the intra-species differences in brain complexity, genomic regulation and behavioral repertoire, which may place limits on the generalizability of rodent-based research findings (Fuccillo et al., 2016).

### Striatal dysfunction in the broader context of neuropsychiatric disease

Given the evidence provided here, I believe that a greater understanding of how normal striatal circuit function is perturbed in the presence of ASD-associated mutations will yield great returns. It is worth noting that many neuropsychiatric disorders demonstrate partially overlapping symptom domains that suggest striatal dysfunction. Obsessive-compulsive disorder is marked by deficiencies in reward processing and behavioral control, while schizophrenia, and major depressive disorder both exhibit profound psychomotor retardation, deficits in attention and decreased goal-directed action. Therefore, a deeper exploration of striatal function, employing current viral and genetic technologies to gain access to discrete components of striatal circuitry, may well shed light on a wide range of neuropsychiatric disorders.

## Author contributions

The author confirms being the sole contributor of this work and approved it for publication.

## Funding

MF is supported by a grant from the NIMH (R00MH099243) and a McCabe Fund Award (University of Pennsylvania).

### Conflict of interest statement

The author declares that the research was conducted in the absence of any commercial or financial relationships that could be construed as a potential conflict of interest.
